# SNAP Participation as a Moderator of Food and Nutrition Security and Combined Cardiometabolic Conditions: A Mixed Regression Approach

**DOI:** 10.3390/nu17030576

**Published:** 2025-02-05

**Authors:** Maha Almohamad, Ruosha Li, Natalia I. Heredia, Jayna M. Dave, Eric E. Calloway, Anjail Sharrief, Shreela V. Sharma

**Affiliations:** 1 Center for Health Equity, Department of Epidemiology, The University of Texas Health Science Center at Houston (UTHealth Houston), School of Public Health, Houston, TX 77030, USA; 2 Department of Neurology, McGovern Medical School at The University of Texas Health Science Center at Houston (UTHealth Houston), Houston, TX 77030, USA; 3 Department of Biostatistics and Data Science, The University of Texas Health Science Center at Houston (UTHealth Houston), School of Public Health, Houston, TX 77030, USA; 4 Department of Health Promotion and Behavioral Sciences, The University of Texas Health Science Center at Houston (UTHealth Houston), School of Public Health, Houston, TX 77030, USA; 5 USDA/ARS Children’s Nutrition Research Center, Department of Pediatrics, Baylor College of Medicine, Houston, TX 77030, USA; 6 Center for Nutrition and Health Impact, Omaha, NE 68154, USA

**Keywords:** hypertension, hyperlipidemia, diabetes, moderation analysis, health disparities, social determinants of health

## Abstract

**Objectives**: To examine the relationships between food security, nutrition security, Supplemental Nutrition Assistance Program (SNAP) participation, and cardiometabolic outcomes, including hypertension, hyperlipidemia, or diabetes, among low-income U.S. individuals. **Methods**: A cross-sectional survey of 486 participants (April–June 2021) assessed food and nutrition security and cardiometabolic outcomes. Mixed-effects logistic regression models adjusted for covariates and included a random effect for state of residence. Moderation analyses evaluated SNAP participation’s impact. **Results**: Very low food security was associated with higher odds of having at least one cardiometabolic condition, such as hypertension, hyperlipidemia, or diabetes (AOR = 1.96; 95% CI: 1.04–3.69; *p* = 0.04). SNAP moderated this relationship (*p*-interaction = 0.007), with non-participants experiencing significantly higher risk. Non-SNAP participants with very low food security had 3.17 (95% CI = 1.17–8.61) times higher odds of having a cardiometabolic condition. Among SNAP participants, very low food security was not significantly associated with having a cardiometabolic condition (OR = 1.62; 95% CI = 0.64–4.13). Higher nutrition security was associated with lower odds of having at least one cardiometabolic condition (AOR = 0.59; 95% CI: 0.41–0.83; *p* = 0.002). **Conclusions**: Nutrition security and SNAP participation mitigate cardiometabolic risks, underscoring their importance in public health interventions.

## 1. Introduction

Food insecurity, defined as the inability to afford nutritionally adequate and safe foods for an active and healthy life, remains a significant public health issue in the United States (U.S.) [1,2]. By August 2023, the number of households experiencing food insecurity had risen dramatically from around 17 million households (12.8%) in 2022 to over 26 million. This crisis significantly affected Hispanic and Black populations, and households with children [1,2,3,4]. Food security encompasses multiple dimensions, including availability, accessibility, utilization, and stability, as well as newer concepts such as agency and sustainability [5,6]. Each of these dimensions is important for determining whether individuals can reliably obtain food. However, these dimensions primarily focus on access and stability rather than the quality of food consumed. Nutrition security, a distinct but related concept, emphasizes consistent access to nutrient-rich, health-promoting foods without resource limitations or worry about food availability. Unlike food security, nutrition security specifically captures the dietary quality and nutritional adequacy necessary for health benefits. Nutrition security can exist independently of food insecurity and may have a distinct influence on health outcomes [7,8,9].

Poor diet quality, often resulting from limited access to nutrient-rich foods, is closely linked to food and nutrition insecurity [9]. Poor diet quality not only stems from limited access to nutrient-rich foods but also includes patterns characterized by a high intake of ultra-processed, nutrient-poor foods, such as sugary beverages, refined grains, and foods high in added sugars, saturated fats, and sodium [10,11]. These dietary patterns significantly increase the risk of cardiometabolic diseases, including hypertension, hyperlipidemia, and diabetes [12].

These conditions are prevalent across the U.S., with nearly half of Americans aged 20 and older affected by hypertension, about 35% affected by hyperlipidemia, and about 11% diagnosed diabetes [13]. Poor diet quality is a modifiable risk factor for these conditions, contributing to increased cardiovascular disease (CVD) risk and associated mortality [14,15,16,17]. Previous studies have established a link between food insecurity and heightened cardiometabolic risk, including a 20% increase in hypertension and a 30% increase in hyperlipidemia risk among food-insecure individuals [18,19,20,21,22]. While prior research has extensively documented the adverse health effects of food insecurity, the relationship between nutrition security and cardiometabolic outcomes remains underexplored. This gap in understanding is crucial, as individuals may have access to sufficient food but still experience poor nutrition security due to the high costs of nutrient-dense foods and the widespread availability of ultra-processed, nutrient-poor options.

The Supplemental Nutrition Assistance Program (SNAP) is a federal government program that provides financial assistance to households with low incomes to supplement their grocery budget, aiming to help them maintain adequate nutrition and health, and reduce food insecurity. While SNAP has demonstrated efficacy in reducing food insecurity, its role in improving nutrition security and reducing cardiometabolic risk is less clear. Evidence suggests that while SNAP participation increases food access, structural barriers, such as the affordability of healthy foods and the prevalence of nutrient-poor option, may limit its impact on nutrition security and health outcomes [23,24,25,26]. SNAP participants often face barriers such as the high cost of healthy foods, which may hinder the program’s ability to improve nutrition security and health outcomes [27,28]. The complex interplay between SNAP, food security, nutrition security, and cardiometabolic outcomes requires further investigation.

This study aims to assess the relationships between food security, nutrition security, SNAP participation, and cardiometabolic outcomes (hypertension, hyperlipidemia, or diabetes) among individuals from low-income households in the U.S. While previous research has established the link between food insecurity and cardiometabolic risk, this study extends the literature by incorporating nutrition security as a key determinant of health. Furthermore, we investigate whether SNAP participation moderates these relationships, providing new insights into the potential role of food assistance programs in shaping both food security and dietary quality. We hypothesized that higher food and nutrition security are inversely associated with the prevalence of cardiometabolic conditions. Additionally, we proposed that SNAP participation significantly moderates the relationships between food and nutrition security and these cardiometabolic outcomes. By addressing these gaps, our study contributes to a more nuanced understanding of the intersection between food policy, nutrition security, and health equity.

## 2. Materials and Methods

### 2.1. Study Design

This study conducted a secondary data analysis using cross-sectional survey data collected by the Center for Nutrition and Health Impact (CNHI) from April to June 2021. The original survey aimed to develop and validate new measures of nutrition security and dietary choice, targeting households at risk for food insecurity across the U.S. By focusing on nutrition security, the survey sought to capture households’ perceived ability to access healthful foods that align with their dietary preferences and needs. Our analysis used these data to explore how levels of food and nutrition security relate to cardiometabolic conditions among low-income adults. The original study was approved by the University of Nebraska Medical Center’s Institutional Review Board (IRB), with data de-identified prior to analysis, and the University of Texas Health Science Center’s (UTHealth Houston) Committee for the Protection of Human Subjects granted an exemption from further review.

### 2.2. Study Population

The study population included adults aged 18 and older from households at risk of food insecurity across five U.S. states: California, Florida, Maryland, North Carolina, and Washington. A total of 486 respondents completed the survey. Participants were recruited through local community organizations, such as food pantries, shelters, and resource centers, using a convenience sampling approach. Participants provided written informed consent, and data were collected through both electronic and paper-based surveys, enabling wider accessibility for respondents. For additional details on the study’s recruitment, data collection procedures, and sample characteristics, refer to Calloway et al. (2022) [8].

### 2.3. Measures

#### 2.3.1. Food Security

Food security status was assessed using the 18-item U.S. Department of Agriculture (USDA) Household Food Security Survey Module (HFSSM) [29]. A score ranging from 0 to 18 was calculated by adding the affirmative responses for households with and without children within a 12-month recall period, with lower scores indicating higher food security. Households were categorized into four levels: high (0 affirmative responses), marginal (1–2 affirmative responses), low (3–5 affirmative responses for households without children; 3–7 affirmative responses for households with children), and very low (6–10 affirmative responses for households without children; 8–18 affirmative responses for households with children) food security.

#### 2.3.2. Nutrition Security

Nutrition security was assessed using a validated four-item scale developed by the CNHI [8]. This scale captures concerns regarding access to and consumption of healthful foods, as well as dietary variety. The scale was designed to complement food security measures by assessing food quality rather than availability alone. Validation of the instrument demonstrated strong internal consistency (Cronbach’s alpha = 0.85) and construct validity when tested in populations at risk for food insecurity [8]. Previous studies, including Almohamad et al. (2024), ref. [30] have utilized this measure to examine the relationship between nutrition security, food security, and diabetes, further supporting its applicability. Scores ranged from 0 to 4, with higher scores indicating greater nutrition security.

#### 2.3.3. Cardiometabolic Outcomes

The outcome variables included self-reported diagnoses of hypertension, hyperlipidemia, and diabetes, each coded as binary variable (yes/no). Diagnosis of cardiometabolic condition was analyzed individually and also as a combined outcome. In this study, a combined outcome variable was created using ‘or’ logic (e.g., ‘yes’ if hypertension or hyperlipidemia or diabetes was present, ‘no’ otherwise) to assess the presence of any cardiometabolic condition. This approach aligns with the broader concept of cardiometabolic health risks, which are often linked to adverse cardiovascular events (MACE) in the literature [31].

#### 2.3.4. SNAP Participation

SNAP participation was assessed as a binary variable (yes/no), based on whether the household reported receiving SNAP benefits in the past year.

#### 2.3.5. Sociodemographic Factors

These included age (continuous in years), gender (male, female), race/ethnicity (categorical: White Non-Hispanic, Latino/Hispanic, Black Non-Hispanic, or Multi-racial/ethnic or another not listed, Asian Non-Hispanic, or Tribal/Indigenous Non-Hispanic), education level (categorical: less than high school, high school diploma or GED, some college, or associate degree or greater), employment status (categorical: not working, retired, disabled, a full-time homemaker/stay-at-home parent, or a full-time student, work in temporary or seasonal job or work year round <30 h per week, or work year-round in a job for 30 or more hours per week), household income (continuous ranging from USD 3000 to USD 63,000), household composition (categorical: presence of children or not), survey mode (categorical: online or paper based), participation in other nutrition assistance programs (e.g., NSLP, WIC, SNAP) (categorical: yes or no), and food pantry use (categorical: yes or no).

### 2.4. Statistical Analysis

Descriptive statistics were calculated for all variables. Frequency distributions of key population demographics were examined by cardiometabolic outcomes. A Pearson Chi-square test was used to examine the association between categorical variables, and a Student *t*-test or Mann–Whitney U test was used to compare the means of two independent groups or continuous variables. Mixed-effects multivariable logistic regression models were used to assess the relationship between food security, nutrition security, and cardiometabolic outcomes, adjusting for sociodemographic factors. Potential confounders were chosen primarily based on established literature and univariable/unadjusted hypothesis testing was conducted to identify important covariates significantly associated with the exposure or outcome at the 0.05 significance level [32]. Finally, interaction terms were added to the model to test whether SNAP participation moderates the association between food security or nutrition security in separate models and cardiometabolic outcomes. Mixed-effects logistic regression models were used to account for the potential state-level variability, with states included as a random effect to ensure the robustness of our findings. Statistical significance was set at a *p*-value of <0.05. All analyses were performed using STATA 15.0 (StataCorp LLC, College Station, TX, USA).

## 3. Results

### Study Population Characteristics

A total of 486 adults were included in the final analysis. The average age was 45 years, with the majority identifying as female (70%) (Table 1). The racial/ethnic distribution of the sample was 40% non-Hispanic White, 21% Hispanic/Latino, 16% non-Hispanic Black, and 13% other races or ethnicities. The mean annual household income was about USD 15,890 with about 58% of the participants not working. Regarding food security, 36% of the participants reported very low food security, 27% reported low food security, 13% reported marginal food security, and 16% were food secure. Among the participants, 33.5% reported having hypertension, 23.5% reported hyperlipidemia, and 23.7% reported diabetes (Table 1).

Multivariable logistic regression analyses revealed that very low food security was significantly associated with higher odds of having at least one cardiometabolic condition, such as hypertension, hyperlipidemia, or diabetes, compared to those with food security (AOR = 1.96; 95% CI: 1.04–3.69; *p* = 0.04) (Table 2). SNAP participation moderated the relationship between food security and the combined outcome of cardiometabolic conditions (*p*-interaction = 0.007). Among non-SNAP participants, those with very low food security had 3.17 times higher odds (95% CI = 1.17–8.61) of having at least one cardiometabolic condition compared to those with food security. In contrast, among SNAP participants, very low food security was not significantly associated with the combined cardiometabolic outcome (AOR = 1.62; 95% CI = 0.64–4.13) (Table 2). Evidence of effect modification was not statistically significant between food security and hyperlipidemia (*p* > 0.05) or food security and hypertension (*p* > 0.05). Nutrition security did not moderate the relationship between food security and the combined outcome of cardiometabolic conditions (*p*-value > 0.05) (Table 2).

Higher nutrition security was significantly associated with lower odds of having at least one cardiometabolic condition, such as hypertension, hyperlipidemia, or diabetes (AOR = 0.59; 95% CI: 0.41–0.83; *p* = 0.002) (Table 3). Finally, SNAP participation did not moderate the association between nutrition security and the combined outcome of cardiometabolic conditions (*p*-interaction > 0.05) (Table 3).

## 4. Discussion

This study explored the relationships between food security, nutrition security, and cardiometabolic outcomes, specifically hypertension, hyperlipidemia, or diabetes among low-income adults, with a particular focus on the moderating role of SNAP participation. The findings underscore the critical role of nutrition security in influencing cardiometabolic health and provide valuable insights into how socioeconomic factors and program participation may affect these relationships. Our previous research identified a significant relationship between food security, nutrition security, and diabetes, highlighting the role of SNAP participation [30]. Understanding the healthcare implications of addressing individual cardiometabolic conditions, considering their combined impact, is crucial for healthcare providers managing patients with elevated risk profiles. This approach can guide interventions that address the cumulative burden of cardiometabolic risks in vulnerable populations.

The findings suggest that SNAP participation may buffer the negative effects of very low food security on cardiometabolic conditions through multiple mechanisms. Among non-SNAP participants, very low food security significantly increased the odds of having at least one cardiometabolic condition, whereas this association was not observed among SNAP participants. One potential explanation is that SNAP participation may provide protective benefits by alleviating financial and dietary constraints, allowing for the purchase of a greater quantity of food. Additionally, SNAP benefits may enable households to allocate more financial resources toward healthcare, prescription medications, and other essentials that contribute to better management of cardiometabolic conditions. SNAP participation may improve health beyond financial relief by alleviating food-related stress, reducing physiological burdens linked to cardiometabolic risk, and promoting healthcare engagement that supports better disease management.

While SNAP participation reduces food insecurity [26,33], this study highlights its limited role in addressing nutrition insecurity. Higher nutrition security was associated with lower odds of having at least one cardiometabolic condition, emphasizing the importance of addressing food quality in public health strategies. Although SNAP participation moderated the relationship between food security and cardiometabolic conditions, it did not significantly impact the relationship between nutrition security and these outcomes, suggesting its effectiveness lies more in addressing food access than improving diet quality. This raises the need for future research to explore why SNAP participation moderates overall risk rather than isolated conditions and to identify additional support for households facing overlapping food and nutrition insecurity [8,34].

Nutrition security, which extends beyond food security and encompasses both food access and quality, is essential for protecting against cardiometabolic diseases, aligning with previous research on the protective role of diet quality [1,19,21]. Future research and public health interventions can better address the root causes of health inequities and improve population health outcomes by prioritizing nutrition security [34]. Many healthcare systems have already integrated food insecurity screenings into electronic medical records [35], which have been crucial in identifying patients at risk of insufficient food access. However, screening solely for food insecurity may overlook important information about a patient’s diet quality and nutrition. There is growing recognition, as emphasized by the CNHI, of the need to expand screenings to include nutrition security, assessing both the quantity and quality of available food. Screening for nutrition security would provide healthcare providers a more comprehensive understanding of patients’ dietary patterns, enabling them to identify individuals at risk for nutrient deficiencies and cardiometabolic conditions more effectively [36]. Beyond screening, developing effective interventions to address food insecurity and nutrition insecurity in clinical care are also needed.

Organizations such as the American Heart Association (AHA) and Feeding America emphasize the need to address nutrition security [37]. The AHA has called for integrating nutrition security screenings into routine practice to better manage patients at risk for poor diet quality, which is closely linked to cardiometabolic diseases [34,38]. Similarly, Feeding America has emphasized the importance of ensuring access to nutrient-rich foods, particularly for vulnerable populations, to prevent chronic disease and improve overall health [37]. Integrating nutrition security assessments into healthcare and public health systems would enable the development of more targeted interventions, providing those with low nutrition security access to healthier food options. This approach would deepen our understanding of the complex relationships between food access, diet quality, and cardiometabolic health, ultimately leading to strategies that improve health [19,39].

### Limitations

This study has several limitations. The cross-sectional design limits causal inferences, and the reliance on self-reported data may introduce recall bias. Additionally, the study sample, while diverse, may not be fully representative of all food-insecure populations in the U.S., particularly given the overrepresentation of females and the geographic focus on five states. Differences in state-level SNAP policies, cost of living, and food access environments may influence the observed relationships, potentially limiting the generalizability of our findings to other regions with distinct socioeconomic conditions. Moreover, the study primarily includes low-income adults, and the results may not extend to populations with higher incomes or different patterns of food assistance program utilization. Future studies should examine these relationships across a wider range of geographic and demographic contexts to assess the robustness of these findings. These factors may limit the generalizability of the findings to the broader U.S. population. Furthermore, the sample size may not have been sufficient to detect significant differences, especially in subgroup analysis, which should be considered when interpreting the results.

There are additional limitations worth noting. The use of convenience sampling may have introduced sampling bias, limiting the ability to generalize findings to a broader population. Furthermore, the lack of information on the duration of SNAP participation or the level of benefits received restricts the ability to assess how these factors may influence food and nutrition security or cardiometabolic outcomes. Finally, the absence of data on other potential confounders, such as pre-existing health conditions, lifestyle factors (e.g., physical activity), and dietary habits, may have affected the ability to fully account for other contributors to cardiometabolic risk in the analyses.

## 5. Conclusions

This study underscores the importance of nutrition security in shaping cardiometabolic outcomes among low-income individuals. While SNAP can be effective in mitigating the risks associated with very low food security, addressing nutrition security is crucial for improving diet quality and reducing cardiometabolic disease burdens. Interventions targeting both food and nutrition security, also within the context of SNAP, could enhance health outcomes for vulnerable populations. Healthcare providers should integrate nutrition security assessments into routine care to help inform nutritional needs of patients and enable targeted interventions addressing both food access and quality. Future research should prioritize longitudinal studies to clarify causal relationships between food and nutrition security and cardiometabolic outcomes, while also examining the influence of SNAP benefit levels, enrollment duration, and nutrition education. Policy recommendations informed by these findings include increasing SNAP benefits, expanding access to nutrient-dense foods, and enhancing nutrition education to ensure SNAP addresses both food quantity and quality, ultimately reducing health disparities and improving health outcomes.

## Figures and Tables

**Table 1:** Participant characteristics by cardiometabolic conditions (hypertension, hyperlipidemia, or diabetes) in the Center for Nutrition and Health Impact pilot survey (April to June 2021).

			Hypertension			Hyperlipidemia			Hypertension, Hyperlipidemia, or Diabetes		
	Overall n = 486		Yes n (%) 163 (33.5)		*t*-Test/ Chi-Square	Yes n (%) 114 (23.5)		*t*-Test/ Chi-Square	Yes n (%) 228 (46.9)		*t*-Test/ Chi-Square
	**n**	**mean (SD)**	**n**	**mean (SD)**	***p* value**	**n**	**mean (SD)**	***p* value**	**n**	**mean (SD)**	***p* value**
**Nutrition Security**	432	2.6 (0.89)	145	2.5 (0.85)	0.11	103	2.6 (0.87)	0.73	205	2.5 (0.9)	**0.009**
**Age**	486	45.1 (14.6)	163	51.1 (14.2)	**0.000**	114	52.2 (14.6)	**0.000**	228	49.6 (14.2)	**0.000**
**Annual Income**	475	15,890.5 (11,505.4)	161	15,680.1 (9557.6)	0.78	114	16,486.8 (10,985.6)	0.53	225	15,700 (10,305.9)	0.732
**Food Security**	**n**	**%**	**n**	**%**	***p* value**	**n**	**%**	***p* value**	**n**	**%**	***p* value**
Food security	83	16.1	28	17.2	**0.000**	21	18.4	**0.000**	37	16.2	**0.000**
Marginal food security	68	13.2	25	15.3		19	16.7		31	13.6	
Low food security	139	26.9	35	21.5		29	25.4		62	27.2	
Very low food security	185	35.8	69	42.3		41	36		91	39.9	
**Gender (%)**											
Male	111	21.5	45	27.6	**0.000**	33	29	**0.000**	52	22.8	**0.000**
Female	364	70.4	114	69.9		80	70.2		172	75.4	
**Race/Ethnicity (%)**											
WNH	207	40	69	42.3	**0.000**	56	49.1	**0.000**	92	40.4	**0.000**
Latino/H	109	21.1	27	16.6		26	22.8		54	23.7	
BNH	85	16.4	41	25.2		14	12.3		45	19.7	
Other	66	12.8	21	12.9		15	13.2		31	13.6	
**Education (%)**											
Less than HS	47	9.1	18	11	**0.000**	12	10.5	**0.000**	27	11.8	**0.000**
HS Diploma/GED	165	31.9	63	38.7		37	32.5		78	34.2	
Some college	119	23	35	21.5		24	21.1		51	22.4	
Associates degree or greater	134	25.9	39	23.9		39	34.2		63	27.6	
**Employment Status (%)**											
Not working	302	58.4	115	70.6	**0.000**	90	79	**0.000**	164	71.9	**0.000**
Part-time/temporary	88	17	24	14.7		17	14.9		32	14	
Full-time	81	15.7	21	12.9		6	5.3		27	11.8	
**Nutrition Assistance Programs (% Yes)**											
NSLP	175	33.9	57	35	**0.000**	33	29	**0.000**	81	35.5	**0.000**
WIC	76	14.7	12	7.4	**0.000**	8	7.0	**0.000**	26	11.4	**0.000**
SNAP	281	54.4	93	57.1	**0.000**	75	66.8	**0.000**	140	61.4	**0.000**
Food Pantry Use	370	71.6	133	81.6	**0.000**	88	77.2	**0.000**	176	77.2	**0.000**
**Household with children—Total**											
None	199	38.5	77	47.2	**0.000**	56	49.1	**0.000**	102	44.7	**0.000**
Yes	287	55.5	86	52.8		58	50.9		126	55.3	
**State**											
California	117	22.6	36	22.1	**0.000**	36	31.6	**0.000**	66	29	**0.000**
Florida	99	19.2	34	20.9		19	16.7		43	18.9	
Maryland	80	15.5	32	19.6		16	14		40	17.5	
North Carolina	85	18.4	30	18.4		20	17.5		35	15.4	
Washington	85	18.4	31	19		23	20.2		44	19.30	
**Survey Mode**											
Online	347	67.1	107	65.6	**0.000**	86	75.4	**0.000**	163	71.5	**0.000**
Paper	139	26.9	56	34.4		28	24.6		65	28.5	

Bolded *p*-values indicates statistical significance at *p* < 0.05.

**Table 2:** Adjusted odds ratios for associations between food security and cardiometabolic conditions (hypertension, hyperlipidemia, or diabetes), stratified by SNAP participation and nutrition security.

		Hypertension			Hyperlipidemia			Hypertension, Hyperlipidemia, or Diabetes		
Subgroup	Food Security ^b^ (n = 475)	AOR 95% CI	*p*	*p*_INT_	AOR 95% CI	*p*	*p*_INT_	AOR 95% CI	*p*	*p*_INT_
**Overall ^a^**										
	Marginal Food Security (n = 68)	1.64 0.74, 3.62	0.22		1.04 0.45, 2.40	0.92		1.41 0.67, 2.97	0.36	
	Low Food Security (n = 139)	0.97 0.47, 2.01	0.94		0.79 0.37, 1.67	0.53		1.39 0.72, 2.70	0.32	
	Very Low Food Security (n = 185)	1.89 0.96, 3.72	0.07		0.92 0.45, 1.88	0.81		1.96 1.04, 3.69	**0.04**	
**By SNAP ^c^** **(n = 486)**				0.26			**0.053**			**0.007**
No (n = 205)										
	Marginal Food Security	1.59 0.49, 5.19	0.44		0.89 0.23, 3.36	0.86		1.43 0.46, 4.41	0.53	
	Low Food Security	0.75 0.23, 2.39	0.63		0.25 0.06, 1.15	0.08		0.75 0.26, 2.21	0.60	
	Very Low Food Security	2.82 0.99, 7.99	**0.051**		1.17 0.37, 3.73	0.79		3.17 1.17, 8.61	**0.02**	
Yes (n = 281)										
	Marginal Food Security	1.83 0.55, 6.10	0.33		1.30 0.39, 4.31	0.67		1.38 0.46, 4.15	0.56	
	Low Food Security	1.23 0.42, 3.58	0.71		1.46 0.51, 4.22	0.48		2.12 0.81, 5.54	0.13	
	Very Low Food Security	1.74 0.62, 4.84	0.29		1.01 0.36, 2.87	0.98		1.62 0.64, 4.13	0.31	
**By Nutrition Security ^d^**				0.25			0.76			0.29

Abbreviations: AOR, Adjusted Odds Ratio; CI, Confidence Interval; SNAP, Supplemental Nutrition Assistance Program; *p*, *p*-value; ***p***_INT_, ***p***_homogeneity/interaction_ value of the interaction term in the multivariable logistic regression model. ^a^ Fully adjusted logistic regression models included age, gender, race/ethnicity, education, employment, NSLP, WIC, SNAP, food pantry use, household with children, and survey mode; random effect by state. ^b^ Reference = food secure. ^c^ Effect modification calculated by SNAP participation. ^d^ Effect modification calculated by nutrition security. Bolded *p*-values indicates statistical significance at *p* < 0.05.

**Table 3:** Adjusted odds ratios for associations between nutrition security and cardiometabolic conditions (hypertension, hyperlipidemia, or diabetes), stratified by SNAP participation.

	Hypertension			Hyperlipidemia			Hypertension, Hyperlipidemia, or Diabetes		
**Subgroup**	AOR 95% CI	*p*	*p*_INT_	AOR 95% CI	*p*	*p*_INT_	AOR 95% CI	*p*	*p*_INT_
Overall ^a^	0.73 0.51, 1.04	0.08		0.69 0.46, 1.02	0.06		0.59 0.41, 0.83	**0.002**	
By SNAP ^b^ (n = 486)			0.16			0.26			0.18
No (n = 205)	0.68 0.36, 1.28	0.23		–			0.56 0.29, 1.08	0.08	
Yes (n = 281)	0.97 0.60, 1.56	0.90		–			0.66 0.42, 1.04	0.07	

Abbreviations: AOR, Adjusted Odds Ratio; CI, Confidence Interval; SNAP, Supplemental Nutrition Assistance Program; ***p***_homogeneity/interaction_, *p* value of the interaction term in the multivariable logistic regression model. ^a^ Fully adjusted logistic regression models included age, gender, race/ethnicity, education, employment, NSLP, WIC, SNAP, food pantry use, household with children, survey mode, and food security status; random effect by state. ^b^ Effect modification calculated by SNAP. Note: Small sample size in SNAP subgroup to detect meaningful differences in individuals with hyperlipidemia. Values are replaced with (–) in the table to indicate insufficient data for reliable estimation or comparison. Bolded *p*-values indicate statistical significance at *p* < 0.05.

## Data Availability

The data presented in the study are openly available on https://www.centerfornutrition.org/food-security-measures (accessed on 5 June 2024).

## Abbreviations

The following abbreviations are used in this manuscript:

SNAP	Supplemental Nutrition Assistance Program
AOR	Adjusted Odds Ratio
CVD	cardiovascular disease
CNHI	Center for Nutrition and Health Impact
USDA	U.S. Department of Agriculture
HFSSM	Household Food Security Survey Module
NSLP	National School Lunch Program
WIC	Special Supplemental Nutrition Program for Women, Infants, and Children
AHA	American Heart Association
HS	high school
WNH	White Non-Hispanic
BNH	Black Non-Hispanic
CI	Confidence Intervals
